# Interaction of C1q With Pentraxin 3 and IgM Revisited: Mutational Studies With Recombinant C1q Variants

**DOI:** 10.3389/fimmu.2019.00461

**Published:** 2019-03-14

**Authors:** Isabelle Bally, Antonio Inforzato, Fabien Dalonneau, Matteo Stravalaci, Barbara Bottazzi, Christine Gaboriaud, Nicole M. Thielens

**Affiliations:** ^1^Université Grenoble Alpes, CEA, CNRS, IBS, Grenoble, France; ^2^Humanitas Clinical and Research Center, Rozzano, Italy; ^3^Department of Biomedical Sciences, Humanitas University, Pieve Emanuele, Italy

**Keywords:** complement C1q, PTX3, IgM, site-directed mutagenesis, molecular interactions, complement activation

## Abstract

Pentraxins and complement defense collagens are soluble recognition proteins that sense pathogens and altered-self elements, and trigger immune responses including complement activation. PTX3 has been shown to interact with the globular recognition domains (gC1q) of the C1q protein of the classical complement pathway, thereby modulating complement activity. The C1q-PTX3 interaction has been characterized previously by site-specific mutagenesis using individual gC1q domains of each of the three C1q chains. The present study is aimed at revisiting this knowledge taking advantage of full-length recombinant C1q. Four mutations targeting exposed amino acid residues in the gC1q domain of each of the C1q chains (Lys^A200^Asp-Lys^A201^Asp, Arg^B108^Asp-Arg^B109^Glu, Tyr^B175^Leu, and Lys^C170^Glu) were introduced in recombinant C1q and the interaction properties of the mutants were analyzed using surface plasmon resonance. All C1q mutants retained binding to C1r and C1s proteases and mannose-binding lectin-associated serine proteases, indicating that the mutations did not affect the function of the collagen-like regions of C1q. The effect of these mutations on the interaction of C1q with PTX3 and IgM, and both the PTX3- and IgM-mediated activation of the classical complement pathway were investigated. The Lys^A200^Asp-Lys^A201^Asp and Lys^C170^Glu mutants retained partial interaction with PTX3 and IgM, however they triggered efficient complement activation. In contrast, the Arg^B108^Asp-Arg^B109^Glu mutation abolished C1q binding to PTX3 and IgM, and significantly decreased complement activation. The Tyr^B175^Leu mutant exhibited decreased PTX3- and IgM-dependent complement activation. Therefore, we provided evidence that, in the context of the full length C1q protein, a key contribution to the interaction with both PTX3 and IgM is given by the B chain Arg residues that line the side of the gC1q heterotrimer, with a minor participation of a Lys residue located at the apex of gC1q. Furthermore, we generated recombinant forms of the human PTX3 protein bearing either D or A at position 48, a polymorphic site of clinical relevance in a number of infections, and observed that both allelic variants equally recognized C1q.

## Introduction

Immune defense relies on the host capacity to identify pathogenic microorganisms and trigger an efficient anti-infectious response while protecting integrity of its own tissues. Pathogen sensing is mediated by constitutive innate immune molecules that are able to identify characteristic pathogen-associated molecular patterns at the surface of microbes, but also potentially noxious elements from self, such as dying cells. Recognition of these cell surface motifs elicits effector mechanisms aimed at containing early infection while instructing appropriate adaptive immune response, and supporting safe removal of apoptotic cell/debris by phagocytes (1, 2). Pentraxins and defense collagens are evolutionarily conserved multimeric pattern recognition proteins that are part of the humoral arm of innate immunity and play a vital role in the first line of anti-microbial defense and in the maintenance of tissue homeostasis (3).

**Figure 1:**
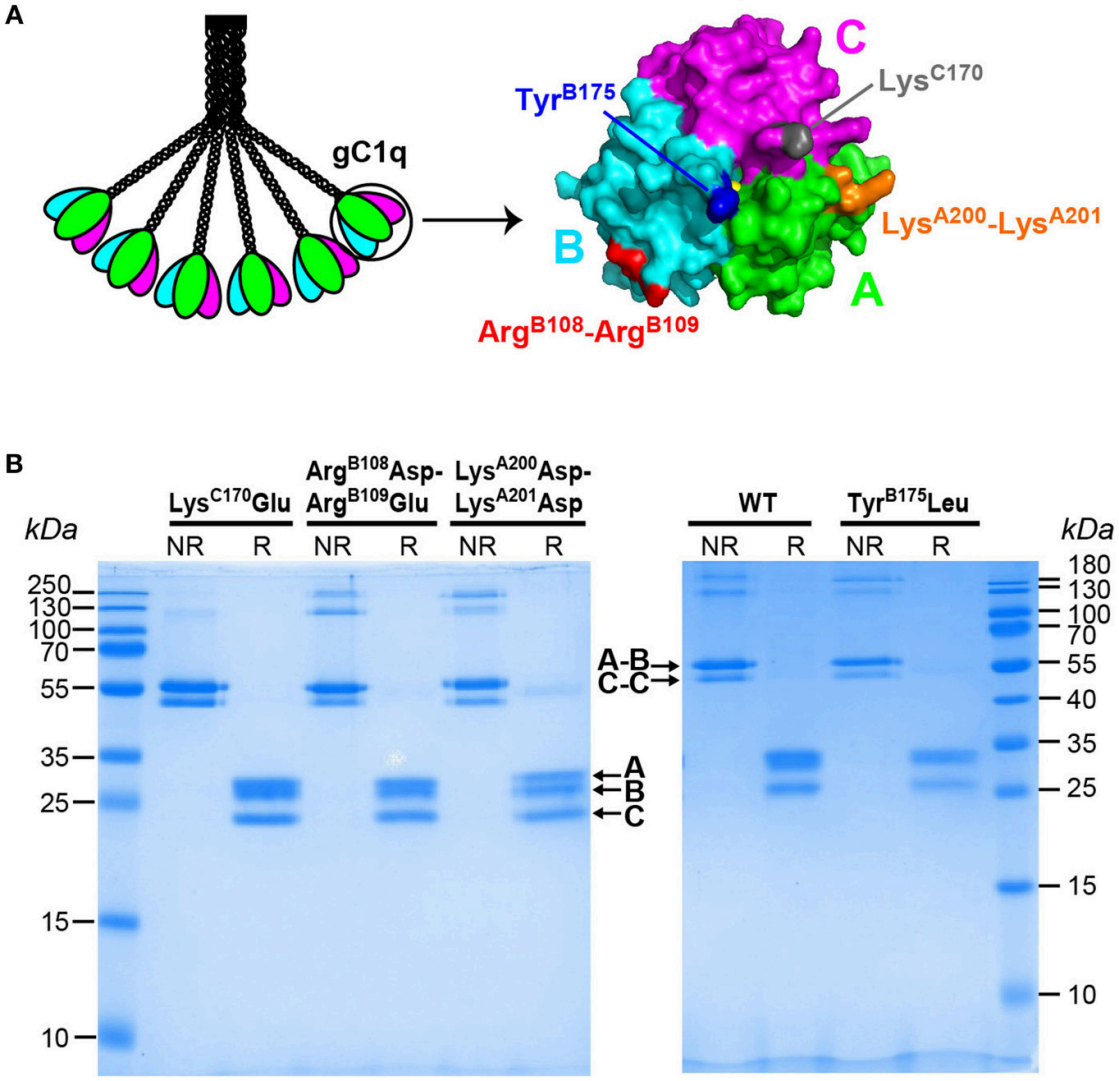
Location of the mutated residues on gC1q structure and SDS-PAGE analysis of the C1q variants. **(A)** Schematic representation of the C1q molecule and 3-D structure of C1q globular domain (gC1q) with C1qA (pink), C1qB (cyan), and C1qC (green). The location of the mutated residues is indicated on the gC1q heterotrimer structure [figure generated using the Mac Pymol software; PDB ID 2wnv (35)]. Tyr^B175^ and Lys^C170^ are located at the apex of the gC1q heterotrimer and Lys^A200^-Lys^A201^ and Arg^B108^-Arg^B109^ are located on the side surfaces of the gC1qA and gC1qB modules, respectively. **(B)** SDS-PAGE analysis and Coomassie blue staining of the C1q variants, under non-reducing (NR) and reducing (R) conditions.

The family of soluble complement defense collagens comprises C1q, collectins including mannose-binding lectin (MBL) and the newly described collectin-10 (CL-K1) and collectin-11 (CL-L1), and the lectin-like proteins ficolins. C1q is the most complex defense collagen since it is composed of 3 homologous yet distinct polypeptide chains A, B, and C that are encoded by three different genes. Each C1q chain comprises an N-terminal collagen-like sequence and a C-terminal globular gC1q module and 18 chains assemble into six heterotrimeric (A-B-C) subunits (4). This hexameric structure exhibits the characteristic shape of a bouquet of flowers, with six collagen-like triple helices (stems), each terminating in a C-terminal globular trimeric head (Figure 1A). Serum C1q circulates in association with a tetramer comprising two copies of each of the homologous C1r and C1s serine proteases. The resulting complex (C1) has the capacity to recognize targets through the globular regions of C1q, which triggers activation of the proteases associated to C1q collagen-like regions and subsequent cleavage of the complement components C4 and C2 (5). The activation fragments C4b and C2a assemble at the target surface to form the C3 convertase of the classical complement pathway that cleaves C3, the central component of the complement system. The classical C3 convertase can also be assembled through activation of the lectin pathway that is initiated by complexes of complement collectins or ficolins and MBL-associated serine proteases (MASPs), which are homologous to C1r and C1s and able to cleave C4 and C2. A third complement activation pathway involves assembly of an alternative C3 convertase containing the C3b fragment and serving to amplify C3 cleavage [reviewed in (6)].

Pentraxins are multimeric proteins with protomer subunits characterized by a conserved C-terminal domain (containing the canonical pentraxin signature HxCxS/TWxS) and assembled into distinctive quaternary structures. The short pentraxins C-reactive protein (CRP) and serum amyloid P component (SAP) are pentameric (7), whereas pentraxin 3 (PTX3), the prototypic long pentraxin, contains an additional N terminal domain and is an octamer composed of two disulfide linked tetramers (8). Pentraxins are acute-phase proteins produced in response to inflammatory stimuli that provide protection against a wide variety of pathogens and participate in the clearance of apoptotic cells (9). PTX3 has regulatory roles in inflammation, where it has been shown to inhibit leukocyte extravasation, and plays additional functions in cancer and tissue repair (10). The three pentraxins have been shown to establish a complex crosstalk with diverse components of complement, thus affecting both the recognition and effector activities of this system. In particular, PTX3 modulates the three complement pathways through interactions with defense collagens (C1q, MBL, ficolin-1, and ficolin-2) and negative regulators of the alternative and classical/lectin C3 convertases, including factor H and C4bp [reviewed in (11)].

Several studies have analyzed single nucleotide polymorphisms (SNPs) in the PTX3 gene. Amongst the 22 SNPs spanning the PTX3 gene (~25 kb) on chromosome 3, three are associated with susceptibility to a number of infections including those mediated by *Aspergillus fumigatus* (12–15), *Mycobacterium tuberculosis* (16) and *Pseudomonas aeruginosa* (17). Two of them are located in intronic regions of the gene (rs2305619 in intron 1, and rs1840680 in intron 2, respectively), and one (missense rs3816527 in exon 2) causes a single amino-acid substitution (p.D48A) at position 48 of the preprotein sequence (i.e., in the N-terminal domain). Epidemiological studies indicate that these three SNPs and the corresponding haplotypes are associated with different plasma levels of the protein, with the D48 exonic allele being enriched in individuals with lower systemic concentrations of PTX3 (18). This information notwithstanding, it is currently unknown whether this exonic polymorphism has qualitative (i.e., functional) in addition to quantitative effects on the crosstalk between PTX3 and the complement system, with major regard to the interaction of this long pentraxin with C1q.

In this regard, binding of C1q to immobilized PTX3 has been reported to trigger complement activation whereas fluid-phase PTX3 interferes with C1q binding to complement activators such as antigen-antibody complexes, in accordance with location of the PTX3 binding site of C1q in the gC1q regions (19). Previous mutagenesis studies on recombinant forms of the gC1qA, gC1qB. and gC1qC domains fused to maltose-binding protein provided initial information on the C1q amino acid residues at the interface of complexes formed with selected ligands, including immunoglobulins (IgG, IgM) and pentraxins (CRP, PTX3) (20–23). These data highlighted the key contribution of electrostatic forces to the interaction of C1q with most of its ligands, and the central role of two residues, Tyr^175^ in gC1qB and Lys^170^ in gC1qC, to recognition of PTX3 (20). We have recently produced the whole human C1q molecule in a recombinant form and demonstrated its structural similarity to serum-derived C1q, as judged from biochemical analysis and electron microscopy imaging. Recombinant C1q functionality was assessed by its capacity to associate with the C1s-C1r-C1r-C1s tetramer, to recognize physiological C1q ligands including IgG and PTX3, and to trigger complement activation (24). Using site-directed mutagenesis, we have also identified two homologous lysine residues in the collagen-like sequences of the B (Lys^61^) and C (Lys^58^) chains of C1q that play a key role in the interaction with C1r and C1s and confirmed that C1q shares with MBL and ficolins a common mechanism of interaction with its associated proteases (24).

The availability of recombinant full-length C1q prompted us to revisit the C1q-PTX3 interaction using site-directed mutagenesis. To this end, we generated four C1q mutants targeting exposed amino acid residues in the gC1q domain of the different chains, including Tyr^B175^ and Lys^C170^ and investigated the impact of these mutations on the C1q-PTX3 interaction and the PTX3-mediated activation of the classical complement pathway. The effect on the interaction of C1q with its canonical ligand IgM was studied in parallel for comparison purposes. Furthermore, we addressed the functional impact of the p.D48A polymorphism on C1q recognition by PTX3.

## Materials and Methods

### Proteins and Reagents

A recombinant form of the human PTX3 protein (with D at position 48) was made in a CHO cell line (25), and used in surface plasmon resonance (SPR) and complement activation experiments (see below). To assess the effect of the rs3816527 (p.D48A) polymorphism on the interaction with C1q in solid phase binding assays (see below), two PTX3 constructs were generated by overlapping PCR site-directed mutagenesis that contained triplets coding either for D or A at position 48. The corresponding recombinant proteins were expressed in and purified from a HEK293 cell line as previously reported (13). Molar concentration of the recombinant PTX3 from both cell lines was estimated using a Mr value of 340,000 (26). Human IgM, bovine serum albumin (BSA) and FLAG peptide were purchased from Sigma-Aldrich. Oligonucleotides were from Eurogentec and restriction and modification enzymes from New England Biolabs. Recombinant human MASP-2 was produced in S2 cells and quantified as described previously (27).

### Production of the Recombinant C1s-C1r-C1r-C1s Tetramer

The recombinant C1s-C1r-C1r-C1s tetramer was produced in the FreeStyle 293 Expression System (Thermo Fisher), using a pcDNA3.1/Neo(+) plasmid encoding human C1r with a Ser637Ala mutation and a C-terminal Strep-tag (kindly provided by A. Amberger and R. Gröbner, Innsbruck Medical University, Austria) and a plasmid encoding human C1s with a C-terminal FLAG epitope. The latter was generated by fusing the FLAG tag (Asp-Tyr-Lys-Asp-Asp-Asp-Asp-Lys) DNA sequence to C1s DNA (amplified using the VentR polymerase and the pFastBac-C1s plasmid (28) as a template) and cloning into a pcDNA3.1/Zeo(+) plasmid. 293-F cells grown in FreeStyle 293 medium were co-transfected with both plasmids using 293fectin and stable transfectants were selected with 400 μg/ml neomycin and 10 μg/ml zeocin (Thermo Fisher). Recombinant C1s-C1r-C1r-C1s was purified from the culture supernatant by chromatography on an anti-FLAG M2 affinity column (Sigma-Aldrich) as described by Bally et al. (24). The tetrameric assembly of the two proteins was assessed by size exclusion chromatography on a Superose 6 Increase 10/300 GL column (GE Healthcare). The molar concentration of the tetramer was estimated using a Mr value of 344,500, as determined by mass spectrometry analyses, and an absorption coefficient (A1%, 1 cm) at 280 nm of 13.45 (29).

### Production of C1q Variants

The Lys^A200^Asp-Lys^A201^Asp, Arg^B108^Asp-Arg^B109^Glu, Tyr^B175^Leu, and Lys^C170^Glu mutations were introduced into the C1qA-, C1qB-, and C1qC-FLAG-containing pcDNA3.1/Neo(+), /Hygro(+), and /Zeo(+) plasmids, respectively, using the QuickChange XL site-directed mutagenesis kit (Agilent Technologies) (24). All constructs were checked by dsDNA sequencing (Eurofins Genomics).

Stable 293-F cell lines producing the individual B and C, A and C, or A and B chains of C1q (24), grown in FreeStyle 293 medium containing the appropriate selection antibiotics and 100 μg/ml ascorbic acid (Sigma-Aldrich), were transfected with the plasmids containing the C1qA Lys^200^Asp-Lys^201^Asp mutation, the C1qB Arg^108^Asp-Arg^109^Glu or Tyr^175^Leu mutation, or the C1qC Lys^170^Glu mutation, respectively, using 293fectin. Stable transfectants producing the three chains were generated following additional selection with 400 μg/ml neomycin (Fisher Scientific), 100 μg/ml hygromycin (Fisher Scientific), or 10 μg/ml zeocin (Sigma-Aldrich), respectively.

Recombinant wild-type (WT) and mutated C1q variants were purified from the stably transfected cell culture supernatants by adsorption on insoluble IgG-ovalbumin aggregates (30) and chromatography on an anti-FLAG M2 affinity column as described previously (24). The molar concentration of the C1q variants was estimated using a Mr of 460,000 and A_1%,1cm_ of 6.8.

### SPR Analyses

Analyses were performed at 25°C using a Biacore 3000 instrument (GE Healthcare). BSA and the C1q variants were diluted in 10 mM sodium acetate at the following concentration and pH: BSA, 25 μg/ml, pH 4.0; C1q variants, 50 μg/ml, pH 4.5 (wild-type) or 4.0 (C1q mutants), and immobilized on CM5 sensor chips (GE Healthcare) using the amine coupling chemistry in 10 mM Hepes, 150 mM NaCl, 3 mM EDTA, 0.005% surfactant P20, pH 7.4. Binding of C1q partners was measured at a flow rate of 20 μl/min in 50 mM Tris-HCl, 150 mM NaCl, 2 mM CaCl_2_, 0.005% surfactant P20, pH 7.4. The specific binding signal was obtained by subtracting the signal over the BSA reference surface. Regeneration of the surfaces was achieved by 10 μl injections of 1 M NaCl, 10 mM EDTA, and, if needed, 10–20 mM NaOH. Kinetic data were analyzed by global fitting to a 1:1 Langmuir binding model for at least five concentrations simultaneously, using the BIAevaluation 3.2 software (GE Healthcare). Buffer blanks were subtracted from the data sets used for kinetic analyses. The apparent equilibrium dissociation constants (*K*_D_) were calculated from the ratio of the dissociation and association rate constants (*k*_d_/*k*_a_). Chi2 values were below 6 in all cases.

### Complement Activation Assays

Microtiter plates (Maxisorp Nunc) were coated with PTX3 (10 μg/ml) or IgM (2 μg/ml) in 10 mM NaHCO_3_, pH 9.6 overnight at 4°C. Wells were incubated for 1 h at 37°C with PBS containing 2% BSA (w/v) and washed with PBS containing 0.05% Tween 20 (PBS-T). C1q-depleted serum (CompTech), diluted 1:25 in 5 mM Na veronal, 145 mM NaCl, 5 mM CaCl_2_, 1.5 mM MgCl_2_, pH 7.5 and reconstituted with the recombinant C1q variants (4 μg/ml) was added to the wells and incubated for 1 h at 37°C. The wells were washed with 5 mM Na veronal, 145 mM NaCl, 5 mM EDTA, pH 7.5 and then a rabbit anti-C4 polyclonal antibody (1:1000 dilution) (Siemens Healthcare Diagnostics) was added to each well and incubated for 1 h at 37°C. After washing with PBS-T and incubation with a peroxidase-conjugated goat anti-rabbit polyclonal antibody (diluted 1:20,000 in PBS-T) (Jackson ImmunoResearch) for 1 h at 37°C, plates were washed with PBS-T and developed with 3,3',5,5'-tetramethylbenzidine (Tebu). The reaction was stopped with 1 N H_2_SO_4_ and absorbance was read at 450 nm. Each assay was performed in duplicate and absorbance values were determined after subtracting blank values obtained in the absence of added C1q. Normal human serum was obtained from the Etablissement Français du Sang Rhône-Alpes (agreement number 14-1940 regarding its use in research). Statistical analysis was performed using a paired two-tailed Student *t*-test (GraphPad software), with statistical significance defined as *P* ≤ 0.05.

### Gel Electrophoresis and Lectin Blotting

Aliquots of purified recombinant PTX3 (either A48 and D48 from HEK293, or D48 from CHO) were run under denaturing conditions on Tris acetate 3–8% (w/v) gels (Thermo Fisher) and 8–18% (w/v) gel cards (GE Healthcare), in the absence and presence, respectively, of dithiothreitol, as reducing agent. Following separation, protein bands were stained either with silver nitrate (ProteoSilver™ Silver Stain Kit, Sigma-Aldrich) or Cy5, according to the electrophoretic apparatus used (XCell SureLock™ Mini-Cell Electrophoresis System, Thermo Fisher, or Amersham WB System, GE Healthcare, respectively).

The oligosaccharides linked to the A48 and D48 variants of PTX3 from HEK293 were probed for linkage and content of terminal residues of sialic acid by lectin staining using the DIG Glycan Differentiation Kit (Roche). Briefly, aliquots of both preparations were resolved by SDS-PAGE on Tris-glycine 10% (w/v) gels under reducing conditions and transferred onto Hybond-C Extra membranes. Following blocking, membranes were incubated with *Maackia amurensis* agglutinin (MAA, that recognizes α(2, 3)-linked sialic acid), and bound lectin revealed according to the manufacturer's instructions (26).

### Solid Phase Binding Assays

Binding of the A48 and D48 variants of PTX3 from HEK293 to C1q was assessed using 96 well Maxisorp plates (Nunc) coated with C1q (purified from human serum; Merck Millipore). All dilutions, incubations, and washes were performed in 50 mM HEPES, 100 mM NaCl, 0.1% (v/v) Tween 20, pH 7.40 (HBS-T). Plates were coated overnight at room temperature with proteins in 20 mM Na_2_CO_3_, pH 9.6. Control wells were incubated with buffer alone and treated as for sample wells. Plates were blocked with 1% (w/v) BSA for 2 h at 37°C, and incubated with the PTX3 proteins for 1 h at 37°C. Bound proteins were detected using a rabbit anti-human PTX3 polyclonal antibody (200 ng/ml) followed by a donkey anti-rabbit IgG HRP-conjugate whole antibody (GE Healthcare) and the 3,3',5,5'-tetramethylbenzidine substrate. Absorbance was read at 450 nm and background from uncoated wells subtracted.

## Results and Discussion

### Generation and Quality Control of the C1q Mutants

Four C1q mutants have been produced, with two mutations targeting residues suggested to participate in the interaction with pentraxins (Tyr^B175^Leu and Lys^C170^Glu), and two mutations targeting exposed basic consecutive residues in the A and B chains (Lys^A200^Asp-Lys^A201^Asp, Arg^B108^Asp-Arg^B109^Glu), proposed to contribute to IgG and/or IgM binding (20, 21, 31). The single mutated residues are located at the apex of the gC1q heterotrimer whereas the tandem lysine and arginine residues are located on the side surface of gC1q (Figure 1A).

The C1q mutants were produced in stably transfected 293-F cells expressing the three C1q chains and the recombinant C1q variants purified from the cell culture supernatants as described for C1qWT. SDS-PAGE analysis of the four purified C1q mutants showed a band pattern similar to that obtained for C1qWT, with characteristic A-B and C-C dimers under non-reducing conditions (Figure 1B, NR lanes) and the three A, B, and C chains under reducing conditions (Figure 1B, R lanes). The minor extra bands above 100 kDa, observed only under non-reducing conditions, likely correspond to multimers of the C chain. This is corroborated by the lower intensity of the bands corresponding to C-C dimers compared to A-B dimers whereas the three chains are of equal intensity under reducing conditions. It should be mentioned that these extra bands are also observed with serum-derived C1q (Figure S1). Negative staining electron microscopy imaging revealed no difference between the wild-type protein and the four mutants, with individual molecules harboring a bouquet-like structure with six globular heads and a central stalk (data not shown), further indicating that the mutations had no impact on the assembly of mutated C1q.

**Figure 2:**
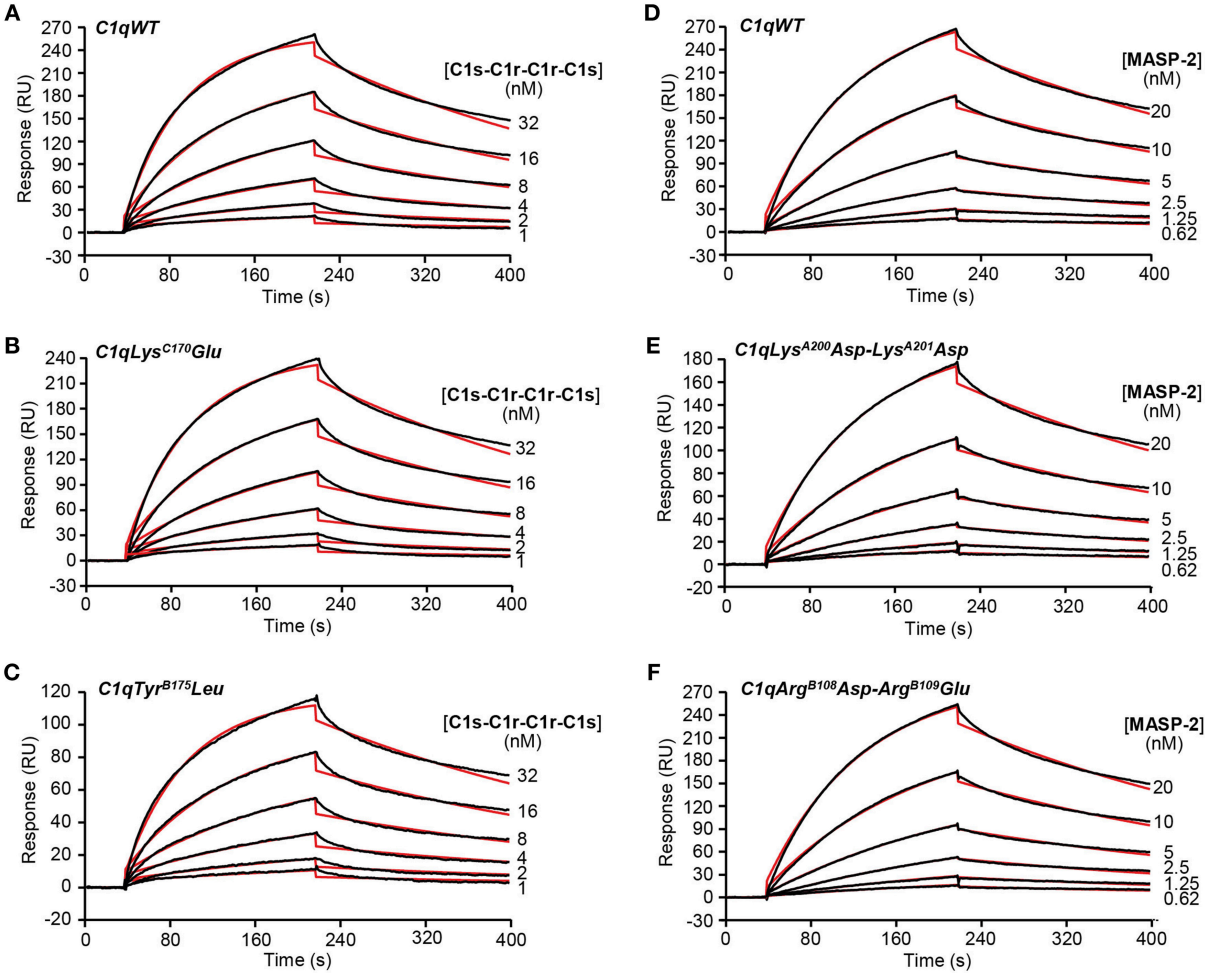
Kinetic analyses of the interaction of the C1s-C1r-C1r-C1s tetramer and the MASP-2 dimer with immobilized C1q variants. Sixty microliter of the C1s-C1r-C1r-C1s tetramer at the indicated concentrations were injected over **(A)** C1qWT (16,300 RU), **(B)** C1qLys^C170^Glu (17,000 RU) and **(C)** C1qTyr^B175^Leu (12,200 RU) in 50 mM Tris-HCl, 150 mM NaCl, 2 mM CaCl_2_, 0.005% surfactant P20, pH 7.4 at a flow rate of 20 μl/min. The MASP-2 dimer was injected over **(D)** C1qWT (13,600 RU), **(E)** C1qLys^A200^Asp-Lys^A201^Asp (13,000 RU), and **(F)** C1qArg^B108^Asp-Arg^B109^Glu (13,700 RU) under the same conditions as in **(A–C)**. The binding signals shown were obtained by subtracting the signal over the BSA reference surface and further subtraction of buffer blanks. Fits are shown as red lines and were obtained by global fitting of the data using a 1:1 Langmuir binding model. Chi2 values were between 0.9 and 5.9. Each kinetic analysis shown is representative of two independent experiments performed on separate sensor chips.

The capacity of the C1q mutants to associate with the C1s-C1r-C1r-C1s tetramer or MASP-2 dimer, the homologous protease of the lectin complement pathway, was analyzed by SPR. The proteases bound to immobilized C1qWT, in accordance with our previous data (24) and to the four C1q mutants (Figure 2). The lower binding level of the C1s-C1r-C1r-C1s tetramer observed for the Tyr^B175^Leu mutant (Figure 2C) can be related to the lower immobilization level of this mutant (12,200 RU) by comparison with C1qWT (16,300 RU, Figure 2A) and the Lys^C170^Glu mutant (17,000 RU, Figure 2B). Kinetic analyses yielded similar binding parameters and dissociation constants for the interaction of the proteases with immobilized C1qWT and the four C1q mutants (Table 1). These data indicated that the mutations in the globular regions did not affect the capacity of the collagen-like regions of the C1q mutants to associate with the C1r/C1r or MASPs proteases.

**Table 1:** Kinetic and dissociation constants for binding of the C1r_2_-C1s_2_ tetramer and MASP-2 dimer to immobilized C1q variants.

**Soluble C1q ligand**	**Constants**	**Immobilized C1q variants**				
		**WT**	**Lys^**C170**^Glu**	**Tyr^**B175**^Leu**	**Lys^**A200**^Asp-Lys^**A201**^Asp**	**Arg^**B108**^Asp-Arg^**B109**^Glu**
C1r_2_-C1s_2_	*k*_a_ (M^−1^ s^−1^)	4.35 ± 0.15 × 10^5^	4.86 ± 0.27 × 10^5^	4.40 ± 0.19 × 10^5^		
	*k*_d_ (s^−1^)	2.88 ± 0.19 × 10^−3^	2.56 ± 0.05 × 10^−3^	2.97 ± 0.04 × 10^−3^		
	*K*_D_ (M)	6.36 ± 0.42 × 10^−9^	5.29 ± 0.40 × 10^−9^	6.75 ± 0.20 × 10^−9^		
MASP-2	*k*_a_ (M^−1^ s^−1^)	4.63 ± 0.12 × 10^5^			4.08 ± 0.06 × 10^5^	4.26 ± 0.05 × 10^5^
	*k*_d_ (s^−1^)	2.34 ± 0.08 × 10^−3^			2.64 ± 0.11 × 10^−3^	2.50 ± 0.13 × 10^−3^
	*K*_D_ (M)	5.04 ± 0.03 × 10^−9^			6.03 ± 0.18 × 10^−9^	5.88 ± 0.31 × 10^−9^

*Values are the means ± SE of two to four separate experiments*.

### PTX3 and IgM Binding Properties of the C1q Variants

**Figure 3:**
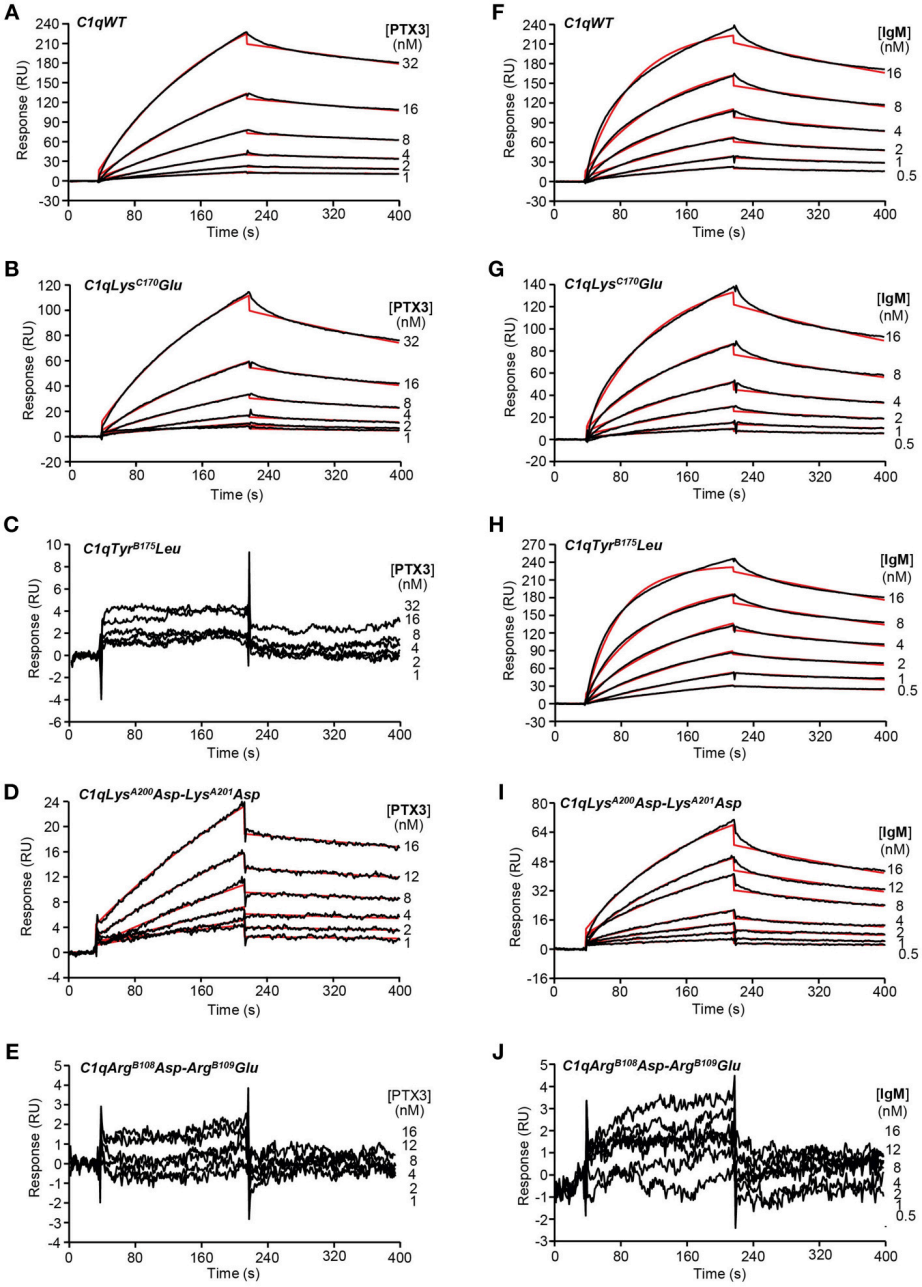
Kinetic analyses of the interaction of PTX3 and IgM with immobilized C1q variants. Sixty microliter of PTX3 at the indicated concentrations were injected over **(A)** C1qWT (16,300 RU), **(B)** C1qLys^C170^Glu (17,000 RU), **(C)** C1qTyr^B175^Leu (12,200 RU), **(D)** C1qLys^A200^Asp-Lys^A201^Asp (18,400 RU), and **(E)** C1qArg^B108^Asp-Arg^B109^Glu (17,500 RU) in 50 mM Tris-HCl, 150 mM NaCl, 2 mM CaCl_2_, 0.005% surfactant P20, pH 7.4 at a flow rate of 20 μl/min. **(F–J)** IgM was injected over the immobilized C1q variants under the same conditions as in **(A–E)**. The binding signals shown were obtained by subtracting the signal over the BSA reference surface and further subtraction of buffer blanks. Fits are shown as red lines and were obtained by global fitting of the data using a 1:1 Langmuir binding model. Chi2 values were between 0.25 and 5.8. Each kinetic analysis shown is representative of two to five independent experiments performed on separate sensor chips.

SPR was further used to investigate the functional impact of the mutations on the interaction of the C1q variants with PTX3 and with IgM, a major complement activating ligand of C1q. The amounts of immobilized C1q ranged from 16,200 to 18,400 RU, except for the Tyr^B175^Leu mutant, for which the immobilization level could not exceed 12,200 RU despite repeated injections. No detectable PTX3 binding was observed for the two C1q variants with mutated B chain residues (Figures 3C,E) whereas the Lys^C170^Glu mutant and the Lys^A200^Asp-Lys^A201^Asp mutant retained the ability of C1qWT to interact with PTX3 (Figures 3B,D), although lower binding levels were observed for the latter mutant (Figures 3A,D). However, kinetic analysis of the interactions yielded *K*_D_ values of the same order, comprised between 5.65 and 10.9 nM (Table 2), even if small differences could be detected between the mutants and C1qWT. For example, the 1.5-fold higher *k*_d_ value for the Lys^C170^Glu mutant may reflect a slightly lower stability of the complex and the 1.5-fold lower *k*_a_ value for the Lys^A200^Asp-Lys^A201^Asp mutant a slightly slower formation of the complex. Comparable effects were observed for binding of both C1q mutants to IgM, with *K*_D_ values ranging from 1.92 to 3.31 nM (Table 2), reflecting a higher apparent affinity for IgM than for PTX3. Interestingly, the immobilized Tyr^B175^Leu mutant retained the ability to interact with IgM (Figure 3H), with even a slightly better affinity (0.89 nM) than C1qWT, arising mainly from a 1.5-fold higher *k*_a_ value. As observed for PTX3 binding, the Arg^B108^Asp-Arg^B109^Glu mutation abolished C1q capacity to interact with IgM (Figure 3J) and the binding levels observed for the Lys^A200^Asp-Lys^A201^Asp mutant were lower than those obtained with C1qWT (Figure 3I).

**Table 2:** Kinetic and dissociation constants for binding of PTX3 and IgM to immobilized C1q variants.

**Soluble C1q ligand**	**Constants**	**Immobilized C1q variants**				
		**WT**	**Lys^**C170**^Glu**	**Tyr^**B175**^Leu**	**Lys^**A200**^Asp-Lys^**A201**^Asp**	**Arg^**B108**^Asp-Arg^**B109**^Glu**
PTX3	*k*_a_ (M^−1^ s^−1^)	1.55 ± 0.21 × 10^5^	1.40 ± 0.01 × 10^5^		9.83 ± 4.08 × 10^4^	
	*k*_d_ (s^−1^)	8.59 ± 0.10 × 10^−4^	1.52 ± 0.11 × 10^−3^	ND	6.34 ± 0.15 × 10^−4^	ND
	*K*_D_ (M)	5.65 ± 0.82 × 10^−9^	1.09 ± 0.07 × 10^−8^		7.84 ± 3.37 × 10^−9^	
IgM	*k*_a_ (M^−1^ s^−1^)	9.19 ± 2.08 × 10^5^	6.81 ± 0.51 × 10^5^	1.47 ± 0.05 × 10^6^	7.17 ± 2.48 × 10^5^	
	*k*_d_ (s^−1^)	1.54 ± 0.19 × 10^−3^	1.67 ± 0.04 × 10^−3^	1.31 ± 0.01 × 10^−3^	2.06 ± 0.22 × 10^−3^	ND
	*K*_D_ (M)	1.92 ± 0.68 × 10^−9^	2.48 ± 0.24 × 10^−9^	8.92 ± 0.41 × 10^−10^	3.31 ± 1.12 × 10^−9^	

*Values are the means ± SE of two to five separate experiments. ND, not determined due to no detectable binding in the concentration range used*.

### PTX3- and IgM-Dependent Complement Activation by the C1q Variants

**Figure 4:**
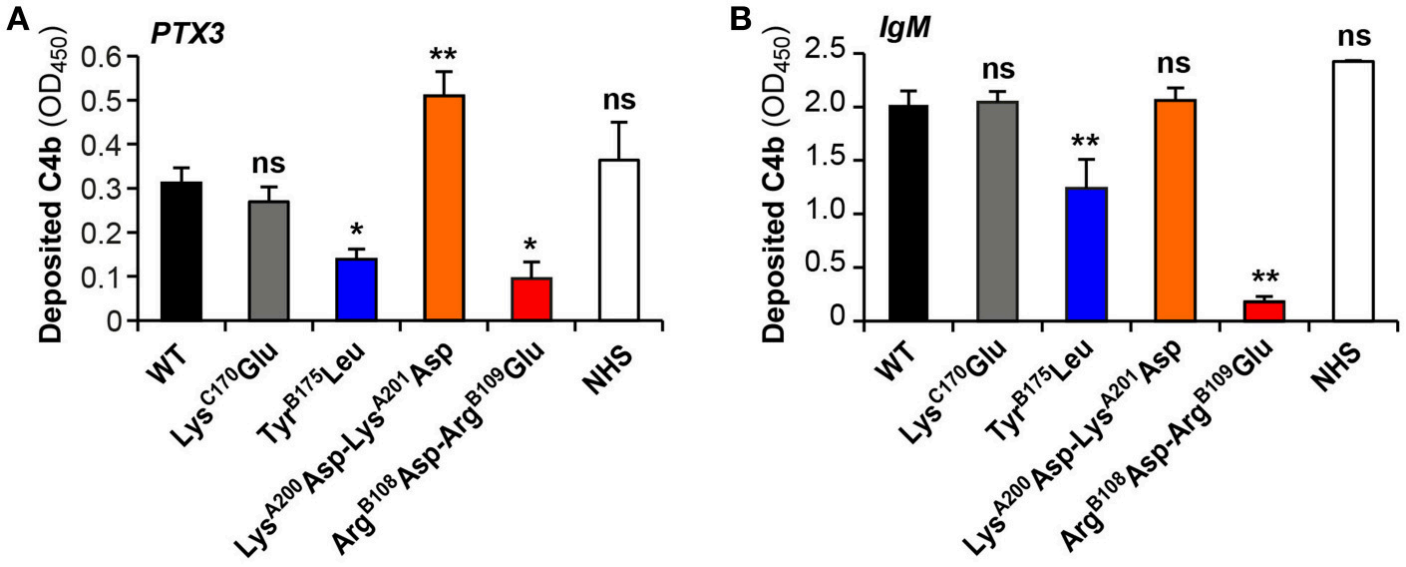
PTX3- and IgM-dependent complement activation by the C1q variants. C1q-depleted serum (1:25 dilution) was reconstituted with the recombinant C1q variants (4 μg/ml) and added to microwells coated with 10 μg/ml PTX3 **(A)** or 2 μg/ml IgM **(B)**. Normal human serum (NHS, 1:25 dilution) was used as a control. The resulting C1-cleaving activity was measured by a C4b deposition assay as described under Material and Methods. Deposited C4b was detected with an anti-human polyclonal antibody, and results are expressed as absorbance at 450 nm (OD_450_), following background subtraction [means ± SEM of three (IgM) and four (PTX3) independent experiments]. Comparisons between C1qWT and each of the mutants or C1q in normal human serum were made using a paired Student *t*-test. ^*^P < 0.05; ^**^P < 0.005; ns, not significant.

The capacity of the C1q variants to trigger complement activation when added to C1q-depleted serum in microwells coated with PTX3 or IgM was analyzed by ELISA. C4b deposition in the wells results from serum C4 cleavage by a functional C1 complex assembled from recombinant C1q and the serum C1r/C1s proteases. As expected, C1qWT yielded amounts of deposited C4b comparable to those obtained with complement-sufficient normal human serum (NHS) in both PTX3 and IgM coated plates. In accordance with the SPR data, no significant difference was observed between the Lys^C170^Glu mutant and C1qWT whereas the Arg^B108^Asp-Arg^B109^Glu mutation strongly decreased both PTX3- and IgM-dependent complement activation (30 and 9% of the signal obtained with C1qWT, respectively) (Figure 4). The Tyr^B175^Leu mutant also exhibited significantly decreased PTX3- and IgM-mediated complement activating capacity (44.5 and 61.4% of the C1qWT value, respectively), in apparent discrepancy with the SPR data that detected no binding of PTX3 (Figure 3C) and a strong interaction with IgM (Figure 3H). As mentioned above, an immobilization level comparable to that of the other mutants (>16,000 RU) could not be reached for the Tyr^B175^Leu mutant (12,200 RU) and the lack of PTX3 binding might be explained by a possible threshold effect. The fact that the interaction with IgM was not affected under the same C1q immobilization conditions might result from a difference in the avidity component of the interactions between hexameric C1q and multivalent PTX3 or IgM. However, it cannot be excluded that the covalent immobilization of this mutant might have influenced its PTX3 binding capacity. In addition, the complement activating assay is performed using coated PTX3 or IgM and the SPR experiments in the reverse configuration (immobilized C1q variants), which might account for the observed discrepancy. Another interesting observation is the fact that the Lys^A200^Asp-Lys^A201^Asp mutant exhibited significantly higher PTX3-dependent complement activating capacity (163% of C1qWT, Figure 4A), which might be linked to the slightly higher stability of the complex observed in SPR experiments.

### The D48 and A48 Allelic Variants of PTX3 and Their C1q Binding Properties

**Figure 5:**
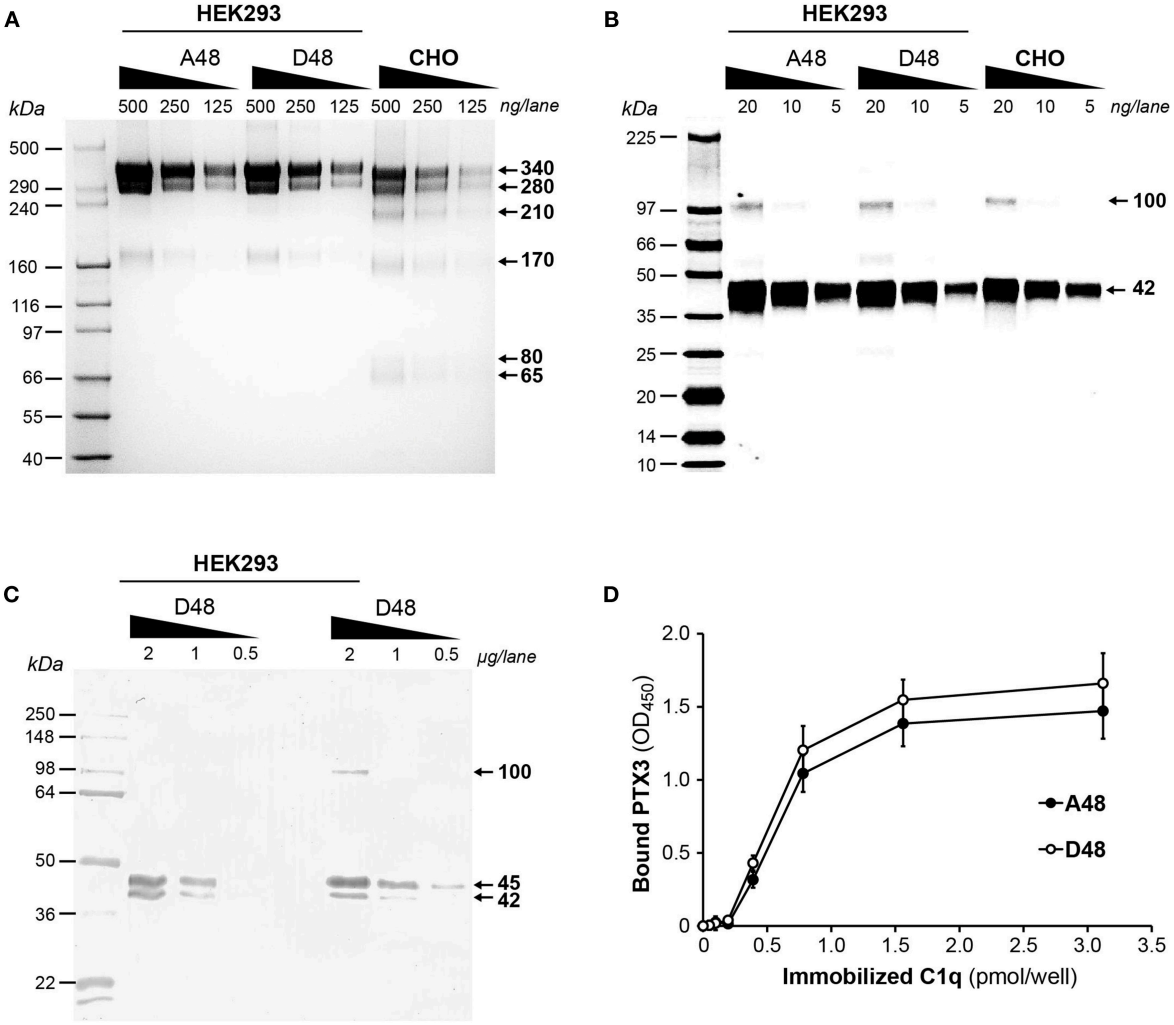
Biochemical characterization of the D48 and A48 allelic variants of PTX3 and their binding to C1q. The indicated amounts of purified recombinant PTX3 (either A48 and D48 from HEK293, or D48 from CHO) were run under denaturing conditions on Tris acetate 3–8% (w/v) gels **(A)** and 8–18% (w/v) gel cards **(B)**, in the absence and presence, respectively, of dithiothreitol. Following separation, protein bands were stained either with silver nitrate **(A)** or Cy5 **(B)**. **(C)** Aliquots of both A48 and D48 preparations from HEK293 were resolved on Tris-glycine 10% (w/v) gels under reducing conditions, transferred onto membranes, and probed with MAA lectin. **(A**–**C)** representative gels from three independent experiments are shown, with molecular mass markers on the left, and apparent molecular mass values observed for the resolved bands on the right. **(D)** The effect of the p.D48A polymorphism on the interaction of PTX3 with C1q was assessed by solid phase binding assay using microwells coated with the indicated amounts of C1q that were incubated with the A48 and D48 variants (both at 3 nM). Bound PTX3 was revealed with an anti-human polyclonal antibody, and results are expressed as absorbance at 450 nm (OD_450_), following background subtraction (three independent experiments performed in quadruplicate, *n* = 12, mean ± SD).

To assess if the exonic polymorphism p.D48A in the PTX3 gene affects the protein's binding to C1q, recombinant forms of the D48 and A48 allelic variants were made in a HEK293 cell line and purified by immunoaffinity chromatography as previously described (13). SDS-PAGE analysis, performed on Tris acetate 3–8% gels under non-reducing conditions (Figure 5A), revealed a major band with an apparent molecular mass of 340 kDa, consistent with PTX3 protomers being mainly assembled into octamers stabilized by disulfide bonds, in both preparations. Additional bands were detected at apparent molecular masses of 280, 210, 170, 80, and 65 kDa, and this pattern was consistent amongst the two allelic variants made in HEK293 and the CHO protein, here used as a reference (8). Upon reduction, a major band at 42 kDa (close to the expected molecular mass for PTX3 monomers, i.e., ~42.5 kDa) and a minor one at 100 kDa (likely corresponding to dimers of the protein, originating from partial reduction) were observed following Cy5 staining in all PTX3 proteins (Figure 5B). Therefore, no significant difference in terms of quaternary structure and homogeneity was noticed amongst the D48 and A48 allelic variants, and the electrophoretic profiles of these proteins under both reducing and non-reducing conditions were comparable to that of the CHO-derived PTX3, taken as a reference. Given that protein glycosylation has been implicated in a number of PTX3 functions in innate immunity and inflammation (32), and, most importantly, sialic acid has been shown to modulate the interaction of PTX3 with C1q (33), we analyzed the sialylation status of the D48 and A48 allelic variants by lectin blotting, using MAA to probe the terminal α(2, 3)-linked sialic acid residues. As shown in Figure 5C, both proteins gave two major MAA-reactive bands at 45 and 42 kDa, indicative of glycoform populations with distinct sialylation (possibly, bi- and tri-antennary complex oligosaccharides), and a minor signal at 100 kDa (likely corresponding to protein dimers, as described in Figure 5B). Therefore, the two recombinant variants of PTX3 were virtually identical in terms of quaternary structure and glycosylation, thus amenable to comparative functional studies. In this regard, when assayed in solid phase binding experiments, the D48 and A48 alleles had comparable binding to plastic-immobilized C1q at each applied concentration (Figure 5D), indicating that in the described experimental conditions the p.D48A polymorphism does not affect the interaction of PTX3 with C1q.

## Conclusion

**Figure 6:**
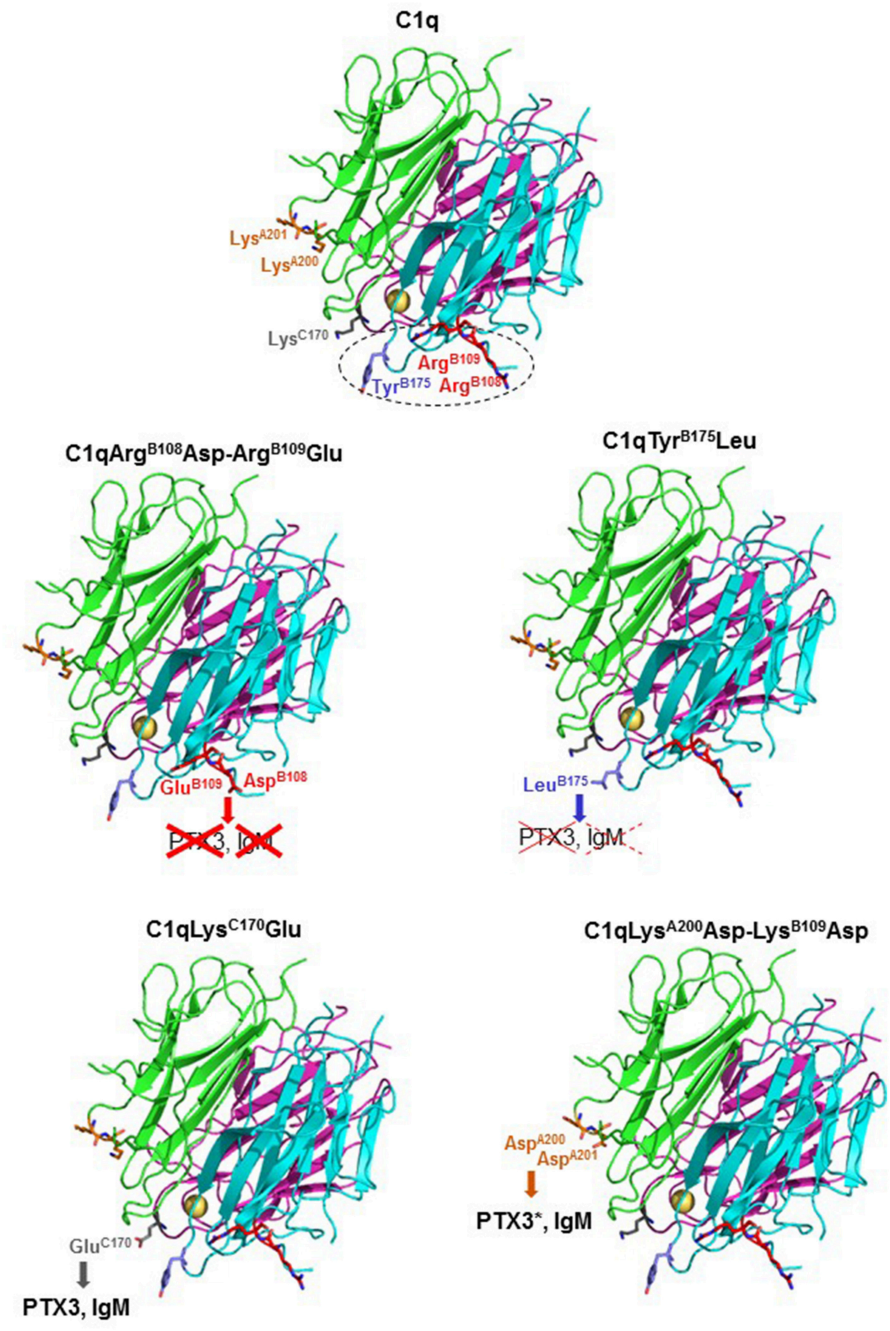
Summary of the C1q mutagenesis results. Ribbon diagrams of the structure of the globular domains of wild-type C1q and of the four mutants are shown. The side chains of the mutated amino acid residues are represented as stick models. The color code for the three C1q chains and the mutated amino acids is the same as in Figure 1. The calcium ion is represented by a yellow sphere. The gC1q region found important for interaction with both PTX3 and IgM and complement activation is delineated by dots. The effects of the C1q mutations toward PTX3 and IgM binding and complement activation are represented as follows: red crosses (solid lines), inhibition of both activities; red crosses (dotted lines), inhibition of complement activation only; bold characters, no significant inhibition. PTX3^*^ indicates that enhancement of PTX3-dependent complement activation was observed.

The present study aimed at revisiting the PTX3-C1q interaction using mutagenesis of full-length recombinant C1q, as compared to the canonical C1q ligand IgM. We confirmed previous observations of an essential role of the B chain residues Arg^108^, Arg^109^, and Tyr^175^ in the interaction with both PTX3 (20) and IgM (21, 34). Our results also suggest no significant contribution of the exposed C1qA Lys^200^ and Lys^201^ in IgM-mediated complement activation, in contrast to previous data obtained with the isolated recombinant gC1qA module showing that the Lys^A200^Glu mutation resulted in a 27% reduction in the binding to solid-phase IgM (21). Intriguingly, the replacement of these two basic residues by acidic residues in the side part of gC1qA significantly enhanced the complement activating capacity of PTX3, which might be related to the slightly better stability of the complex observed in SPR experiments. In a similar way, the C chain Lys^170^Glu mutation had no significant impact on the PTX3 or IgM binding and the complement activating properties of C1q, in contrast to previous observations showing a considerable reduction in gC1qC binding to solid-phase PTX3 (about 40%) (20) and IgM (>30%) (21). These data suggest a differential exposure of Lys^A200^ and Lys^C170^ in the isolated gC1qA/gC1qC modules and in the heterotrimeric globular heads of full-length C1q. Altogether, these mutagenesis results confirm a key electrostatic contribution in the interaction between C1q B chain and PTX3 or IgM (summarized in Figure 6), consistent with the hypothesis that binding of C1q to targets through this region triggers efficient activation of the C1 complex (35, 36).

Given that the exonic polymorphism p.D48A (or rs3816527) in the PTX3 gene forms with rs2305619 in intron 1 and rs1840680 in intron 2 an haplotypic block that has been linked to the susceptibility to selected infections as well as different circulating levels of the protein, it was important to assess whether this amino acid substitution had any effect on C1q recognition. To this end, we generated recombinant forms of the A48 and D48 alleles that were comparable in quaternary structure, homogeneity and sialylation status, thus being amenable to comparative studies. These preparations had similar binding to C1q, which indicates that the p.D48A polymorphism does not alter the C1q binding properties of PTX3, at least in the applied experimental conditions. In addition, we have previously shown that the C-terminal domain of PTX3 mostly mediates the interaction of the long pentraxin with C1q (25), and this is modulated by protein glycosylation (at Asn^220^ in the C-terminal domain). Molecular dynamics simulations suggest that the PTX3 oligosaccharides (via their terminal residues of sialic acid) are in contact with polar amino acids on the solvent exposed surface of the C-terminal domain (33). Given the prominent electrostatic nature of the PTX3-C1q interaction, as supported by our study and previous evidence (20), it is tempting to speculate that these residues are involved in C1q recognition. Further studies are needed to challenge this hypothesis and identify the PTX3 residues that support C1q binding. Finally, the novel C1q mutants generated in this study should allow further exploration of the molecular bases of C1q binding versatility in different physiological contexts.

## Data Availability

The datasets generated for this study are available on request to the corresponding author.

## Author Contributions

NT and CG designed the study. IB, NT, MS, and AI performed the research. IB, CG, AI, BB, and NT analyzed the data. FD, AI, and BB contributed key reagents. NT wrote the manuscript draft. All authors revised and approved the final version of the manuscript.

### Conflict of Interest Statement

Pending patent application by NT and IB: Method for preparing C1q recombinant protein (WO2014 057437). The remaining authors declare that the research was conducted in the absence of any commercial or financial relationships that could be construed as a potential conflict of interest.

## Abbreviations

gC1q: globular domain of C1q
MAA: *Maackia amurensis* agglutinin
MASP: MBL-associated serine protease
MBL: mannose-binding lectin
SPR: surface plasmon resonance.
