# Supplementary material for: Interaction of C1q With Pentraxin 3 and IgM Revisited: Mutational Studies With Recombinant C1q Variants
Source: Front Immunol. 2019 Mar 14;10:461. doi: 10.3389/fimmu.2019.00461 (PMC6426777; doi:10.3389/fimmu.2019.00461)
Supplement: Supplementary file 1 [file Data_Sheet_1.PDF]

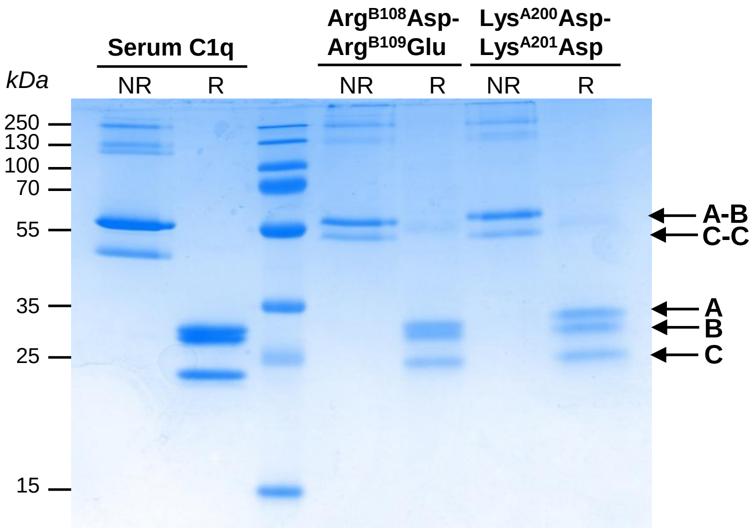

Figure S1. SDS-PAGE analysis and Coomassie Blue staining of purified serum derived C1q and of two C1q mutants generated for this study. NR, non-reducing conditions; R, reducing conditions. High molecular weight bands are observed under non-reducing conditions for the three samples It can be noticed that the C chain of recombinant C1q migrates slightly slower that its counterpart in serum C1q because of the presence of the Flag-Tag.
